# Supplementary material for: Variability in Infants' Functional Brain Network Connectivity Is Associated With Differences in Affect and Behavior
Source: Front Psychiatry. 2021 Jun 9;12:685754. doi: 10.3389/fpsyt.2021.685754 (PMC8220897; doi:10.3389/fpsyt.2021.685754)
Supplement: Supplementary file 1 [file Data_Sheet_1.docx]

**Variability in functional brain network connectivity is associated with differences in newborn infants’ affect and behavior**

*Supplementary Materials*

Caroline M. Kelsey^1,2^, Katrina Farris^2,3^ & Tobias Grossmann^2,4^

^1^ Department of Pediatrics, Division of Developmental Medicine, Boston Children’s Hospital,

Boston, MA, USA

^2^ Department of Psychology, University of Virginia, Charlottesville, VA, USA

^3^ Department of Psychology, Georgia State University, Atlanta, GA, USA

^4^ Max Planck Institute for Human Cognitive and Brain Sciences, Leipzig, Germany

Correspondence:

Tobias Grossmann

Department of Psychology

PO BOX 400400

University of Virginia

Charlottesville, VA 22904

Tg3ny@virginia.edu

**Supplementary Results**

*Regulation/Orienting.* A multiple linear regression using the entry method was conducted with the fNIRS session covariates (alertness, amount of usable data) and four network types (FPN, DMN, HIN, control) as the predictors and regulation/orienting as the outcome variable. The regression model was statistically significant, *F*(6, 66) = 3.16, *p* = .009, *R^2^* = .22. More specifically, connectivity in the DMN was negatively associated with regulation/orienting (*B* = -.94, *SE* = .46, *p* = .043); whereas, connectivity in the FPN was positively associated with regulation/orienting (*B* = .95, *SE* = .39, *p* = .016). Neither the HIN nor the Control network were related to regulation/orienting, all *p-values* > .081.

*Negative Emotionality.* A multiple linear regression using the entry method was conducted with the fNIRS session covariates (alertness, amount of usable data) and four network types (FPN, DMN, HIN, control) as the predictors and negative emotionality as the outcome variable. The regression model was statistically significant, *F*(6, 65) = 2.80, *p* = .018, *R^2^* = .205. More specifically, we found a significant positive relation between HIN connectivity and negative emotionality, (*B* = 2.33, *SE* = .81, *p* = .006). However, none of the other networks (functional nor control) were related to Negative Emotionality, all *p-values* > .15.

*Surgency/Positive Emotionality.* A linear regression was conducted with the four network types (FPN, DMN, HIN, control) predicting surgency/positive emotionality using the entry method. Here, the regression model was not statistically significant, *p* = .40. Moreover, none of the network types were significantly associated with surgency/positive emotionality (all *p*’s > .32).

**Descriptive statistics for channels**

*Supplementary Table 1*. Descriptive statistics for functional connections across networks. Note, associations that survive bonferonni corrections are in bold.

|  |  | oxyHb | | | deoxyHb | | |
| --- | --- | --- | --- | --- | --- | --- | --- |
| Channels | N | Mean | SD | Sig. | Mean | SD | Sig. |
| HIN |  |  |  |  |  |  |  |
| 1--26 | 62 | **0.16** | **0.36** | **0.001** | 0.16 | 0.50 | 0.017 |
| 2--28 | 65 | **0.28** | **0.46** | **< .001** | **0.17** | **0.41** | **0.001** |
| 3--34 | 71 | **0.45** | **0.56** | **< .001** | **0.33** | **0.56** | **< .001** |
| 4--29 | 53 | 0.06 | 0.28 | 0.149 | 0.01 | 0.23 | 0.65 |
| 5--31 | 49 | 0.09 | 0.30 | 0.031 | 0.02 | 0.27 | 0.596 |
| 6--38 | 71 | 0.10 | 0.35 | 0.016 | 0.11 | 0.30 | 0.004 |
| 7--32 | 45 | 0.03 | 0.27 | 0.476 | 0.14 | 0.32 | 0.005 |
| 9--35 | 64 | 0.14 | 0.37 | 0.004 | **0.17** | **0.33** | **< .001** |
| 12--30 | 52 | 0.06 | 0.30 | 0.157 | < .001 | 0.24 | 0.921 |
| 13--36 | **71** | **0.29** | **0.48** | **< .001** | **0.23** | **0.40** | **< .001** |
| 14--39 | 68 | 0.12 | 0.35 | 0.006 | 0.12 | 0.37 | 0.01 |
| 15--45 | 62 | 0.07 | 0.32 | 0.106 | 0.14 | 0.39 | 0.006 |
| 16--33 | **71** | **0.26** | **0.36** | **< .001** | **0.24** | **0.38** | **< .001** |
| 17--40 | 72 | 0.10 | 0.33 | 0.01 | 0.11 | 0.33 | 0.007 |
| 18--48 | 68 | 0.11 | 0.40 | 0.02 | 0.06 | 0.37 | 0.161 |
| 19--37 | 70 | 0.01 | 0.26 | 0.677 | 0.02 | 0.34 | 0.593 |
| 20--44 | 62 | 0.07 | 0.33 | 0.1 | 0.03 | 0.32 | 0.487 |
| 21--46 | 59 | 0.07 | 0.32 | 0.116 | -0.05 | 0.30 | 0.226 |
| 22--41 | 67 | 0.07 | 0.29 | 0.052 | 0.05 | 0.36 | 0.25 |
| 23--47 | 64 | 0.05 | 0.30 | 0.202 | 0.02 | 0.32 | 0.638 |
| 24--49 | 57 | 0.08 | 0.35 | 0.104 | 0.08 | 0.37 | 0.119 |
| FPN |  |  |  |  |  |  |  |
| 2--7 | **70** | **0.18** | **0.37** | **< .001** | 0.07 | 0.37 | 0.114 |
| 2--16 | **71** | **0.20** | **0.42** | **< .001** | 0.08 | 0.42 | 0.112 |
| 3--7 | **69** | **0.19** | **0.39** | **< .001** | 0.05 | 0.35 | 0.261 |
| 3--16 | **70** | **0.20** | **0.41** | **< .001** | 0.12 | 0.43 | 0.025 |
| 7--13 | 69 | 0.11 | 0.41 | 0.03 | 0.12 | 0.37 | 0.01 |
| 13--16 | 68 | 0.06 | 0.42 | 0.229 | 0.10 | 0.42 | 0.059 |
| 28--32 | 41 | 0.02 | 0.21 | 0.631 | 0.07 | 0.25 | 0.074 |
| 28--33 | 65 | **0.28** | **0.43** | **< .001** | 0.14 | 0.44 | 0.013 |
| 32--34 | 43 | -0.06 | 0.28 | 0.177 | 0.06 | 0.26 | 0.165 |
| 32--36 | 46 | -0.04 | 0.26 | 0.306 | 0.01 | 0.25 | 0.782 |
| 33--34 | 72 | **0.49** | **0.53** | **< .001** | **0.42** | **0.47** | **< .001** |
| 33--36 | 74 | **0.47** | **0.50** | **< .001** | **0.49** | **0.54** | **< .001** |
| DMN |  |  |  |  |  |  |  |
| 10--18 | 66 | 0.11 | 0.34 | 0.009 | 0.09 | 0.40 | 0.075 |
| 10--22 | 66 | 0.11 | 0.40 | 0.023 | **0.17** | **0.37** | **0.001** |
| 10--23 | 66 | 0.14 | 0.41 | 0.006 | 0.13 | 0.45 | 0.02 |
| 10--24 | 59 | 0.02 | 0.38 | 0.685 | 0.09 | 0.31 | 0.029 |
| 10--41 | 61 | 0.08 | 0.41 | 0.158 | 0.09 | 0.44 | 0.104 |
| 10--47 | 60 | 0.11 | 0.42 | 0.046 | 0.01 | 0.45 | 0.89 |
| 10--48 | 63 | 0.10 | 0.41 | 0.053 | 0.03 | 0.47 | 0.57 |
| 10--49 | 62 | 0.11 | 0.37 | 0.025 | 0.12 | 0.33 | 0.006 |
| 18--42 | 69 | 0.08 | 0.32 | 0.036 | 0.03 | 0.34 | 0.512 |
| 18--43 | 65 | -0.01 | 0.35 | 0.78 | 0.03 | 0.33 | 0.499 |
| 22--42 | 70 | 0.02 | 0.31 | 0.661 | 0.08 | 0.35 | 0.056 |
| 22--43 | 65 | 0.08 | 0.32 | 0.048 | 0.07 | 0.34 | 0.084 |
| 23--42 | 69 | 0.05 | 0.27 | 0.107 | 0.05 | 0.31 | 0.193 |
| 23--43 | 64 | 0.07 | 0.30 | 0.053 | 0.09 | 0.37 | 0.067 |
| 24--42 | 60 | 0.14 | 0.34 | 0.003 | 0.09 | 0.36 | 0.052 |
| 24--43 | 57 | 0.05 | 0.33 | 0.264 | 0.08 | 0.35 | 0.09 |
| 41--42 | 67 | **0.55** | **0.54** | **< .001** | **0.36** | **0.56** | **< .001** |
| 41--43 | 62 | **0.41** | **0.61** | **< .001** | **0.40** | **0.60** | **< .001** |
| 42--47 | 63 | **0.23** | **0.40** | **< .001** | **0.33** | **0.44** | **< .001** |
| 42--48 | 67 | 0.13 | 0.41 | 0.009 | 0.15 | 0.40 | 0.003 |
| 42--49 | 66 | **0.19** | **0.47** | **0.001** | **0.20** | **0.46** | **0.001** |
| 43--47 | 61 | 0.09 | 0.39 | 0.086 | 0.11 | 0.38 | 0.027 |
| 43--48 | 64 | 0.08 | 0.35 | 0.07 | 0.14 | 0.47 | 0.022 |
| 43--49 | 63 | **0.40** | **0.54** | **< .001** | **0.40** | **0.49** | **< .001** |
| Control |  |  |  |  |  |  |  |
| 15--41 | 68 | 0.07 | 0.39 | 0.167 | 0.07 | 0.36 | 0.108 |
| 15--48 | 70 | 0.08 | 0.38 | 0.097 | 0.06 | 0.45 | 0.3 |
| 15--49 | 69 | 0.13 | 0.38 | 0.006 | 0.11 | 0.38 | 0.016 |
| 18--37 | 70 | -0.02 | 0.32 | 0.693 | 0.03 | 0.31 | 0.381 |
| 18--45 | 61 | 0.06 | 0.37 | 0.197 | 0.05 | 0.32 | 0.257 |
| 18--46 | 63 | 0.04 | 0.32 | 0.373 | 0.09 | 0.36 | 0.049 |
| 19--41 | 66 | 0.04 | 0.31 | 0.326 | 0.12 | 0.33 | 0.004 |
| 19--48 | 68 | 0.10 | 0.29 | 0.004 | 0.08 | 0.40 | 0.086 |
| 19--49 | 67 | 0.09 | 0.29 | 0.012 | 0.07 | 0.36 | 0.093 |
| 21--41 | 65 | 0.01 | 0.31 | 0.779 | -0.03 | 0.30 | 0.448 |
| 21--48 | 66 | 0.02 | 0.31 | 0.532 | 0.03 | 0.37 | 0.545 |
| 21--49 | 65 | 0.06 | 0.30 | 0.138 | 0.09 | 0.32 | 0.032 |
| 22--37 | 70 | 0.02 | 0.30 | 0.648 | 0.00 | 0.30 | 0.911 |
| 22--45 | 60 | **0.13** | **0.29** | **0.001** | **0.13** | **0.32** | **0.002** |
| 22--46 | 62 | 0.07 | 0.24 | 0.033 | -0.02 | 0.33 | 0.657 |
| 24--37 | 60 | 0.03 | 0.39 | 0.614 | 0.02 | 0.26 | 0.527 |
| 24--45 | 53 | -0.01 | 0.33 | 0.795 | 0.10 | 0.28 | 0.015 |
| 24--46 | 54 | 0.12 | 0.35 | 0.012 | 0.03 | 0.28 | 0.421 |

**Covariate Associations**


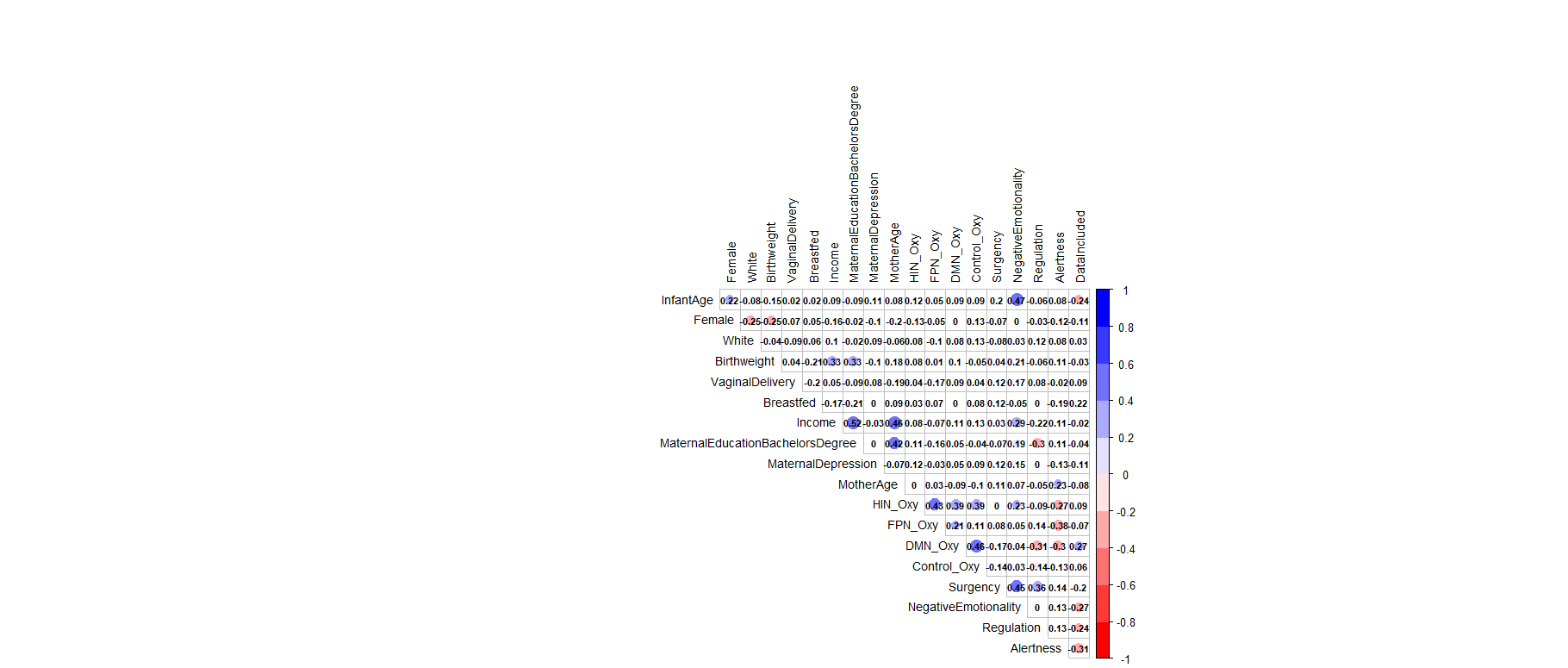


*Supplementary Figure 1.* Spearman’s correlations between socio-demographic factors and study variables. Note, significant associations (*p* < .05) are marked by having a colorful circle in the background.

**Correlations between networks (OxyHb and DeoxyHb)**


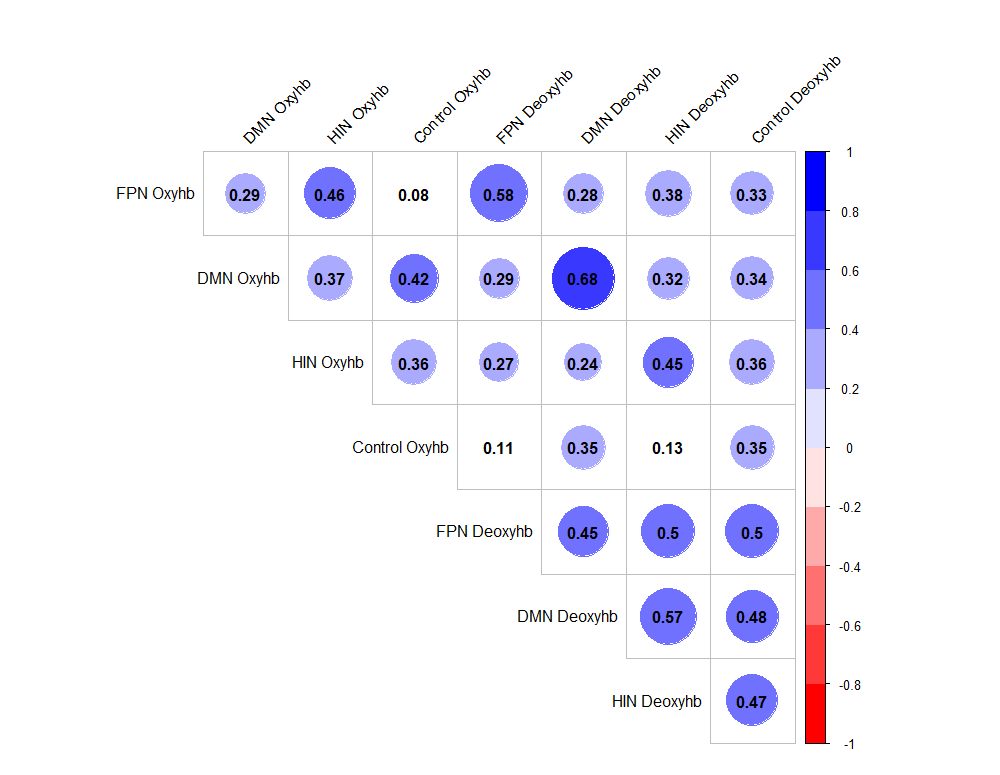


*Supplementary Figure 2.* Pearson correlations between functional networks. Note, significant associations (*p* < .05) are marked by having a colorful circle in the background.


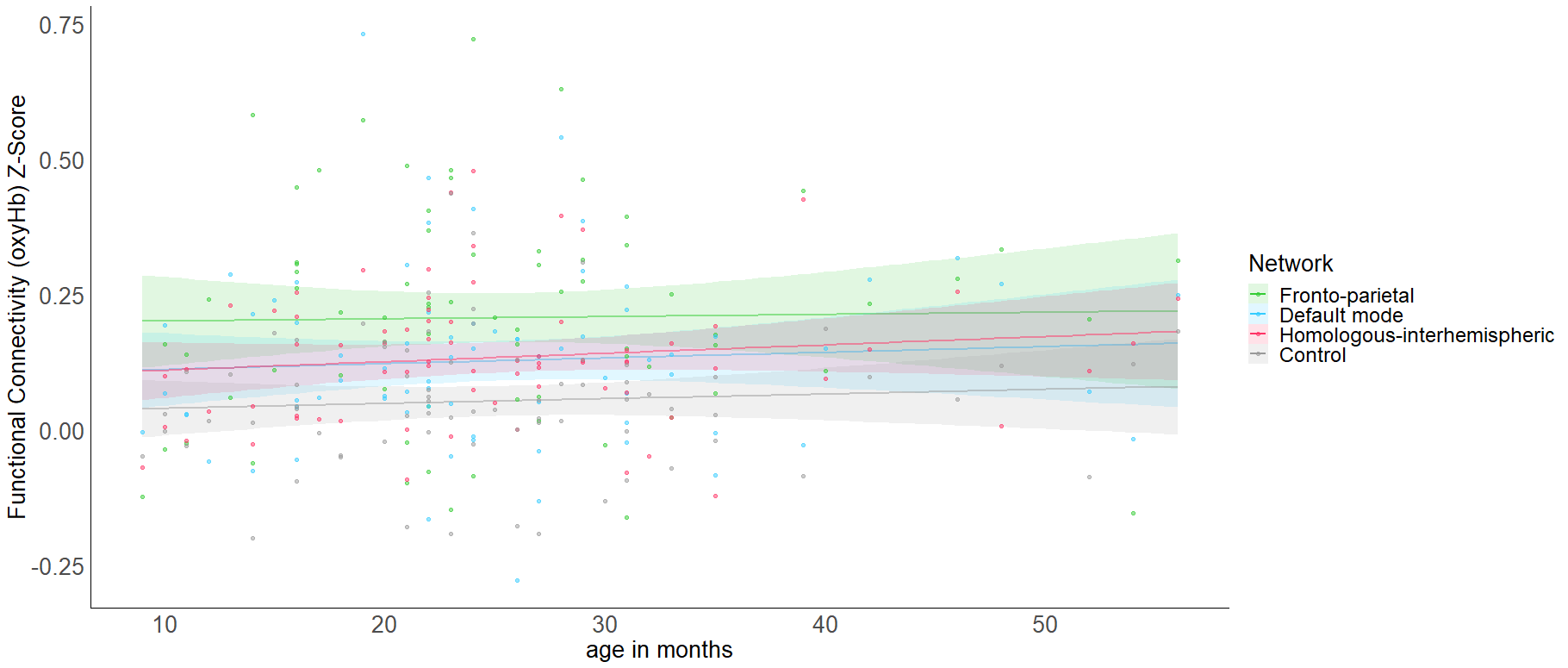


*Supplementary Figure 3.* Depiction of connectivity ranges by infants’ age in months. Note, there are no significant associations between age and functional connectivity levels in any of the networks.

**DeoxyHb Results**

**Functional connectivity (deoxyHb) across networks.**

To analyze differences in overall connectivity levels across networks for deoxyHb an omnibus repeated measures ANOVA with network type (homologous-interhemispheric network [HIN], default mode network [DMN], fronto-parietal network [FPN], control) as a within-subjects factor was conducted. This analysis revealed a significant within-subjects effect across network types, *F*(3, 222) = 11.77, *p* < .001, η^2^ = .137. Post-hoc analyses with Bonferroni adjustments for multiple comparisons were conducted to assess which networks significantly differed from one another. Here, we found that control network (*M* = .05; *SD* = .12) had significantly lower connectivity than the FPN (*M* = .16; *SD* = .20), *p* < .001, HIN (*M* = .11; *SD* = .12), *p* = .001, and the DMN (*M* = .13; *SD* = .16), *p* < .001. However, there was no significant difference found between the functional networks of interest, all *p’s >* .08 (see *Supplementary Figure 4* for more information).


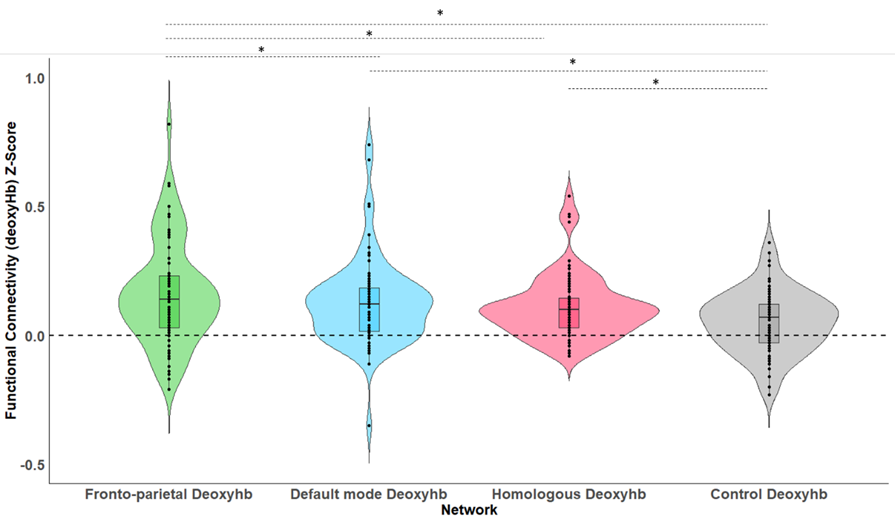


*Supplementary Figure 4.* Shows the average levels of functional connectivity (deoxyHb) and range of variability for each network. The boxplot horizontal lines from bottom to top reflect values for the lower quartile, median, and upper quartile respectively. Note, * *p* < .05.

**Functional connectivity (deoxyHb) and temperament.**

In order to assess how functional connectivity patterns for deoxyHb differentially predicted temperament characteristics, three separate regressions with all four network types (HIN, DMN, FPN, control) predicting each of the three domains of temperament (negative emotionality, regulation/orienting, surgency/positive Emotionality) were conducted.

*Regulation/orienting.* A linear regression was conducted with the four network types (HIN, DMN, FPN, control) predicting regulation/orienting using the entry method. Here, the regression model did not significantly predict regulation/orienting, *p* = .59.

*Negative emotionality.* A linear regression was conducted with the four network types (HIN, DMN, FPN, control) predicting negative emotionality using the entry method. Here, the model did not significantly predict negative emotionality, *p* = .24.

*Surgency/positive emotionality.* A linear regression was conducted with the four network types (HIN, DMN, FPN, control) predicting surgency/positive emotionality using the entry method. Again, the regression model did not significantly predict surgency/positive emotionality, *p* = .67.
